# A decoupled, modular and scriptable architecture for tools to curate data platforms

**DOI:** 10.1093/bioinformatics/btab233

**Published:** 2021-04-08

**Authors:** Momo Langenstein, Henning Hermjakob, Manuel Bernal Llinares

**Affiliations:** European Bioinformatics Institute (EMBL-EBI), European Molecular Biology Laboratory, Wellcome Genome Campus, Cambridge CB10 1SD, UK; European Bioinformatics Institute (EMBL-EBI), European Molecular Biology Laboratory, Wellcome Genome Campus, Cambridge CB10 1SD, UK; European Bioinformatics Institute (EMBL-EBI), European Molecular Biology Laboratory, Wellcome Genome Campus, Cambridge CB10 1SD, UK

## Abstract

**Motivation:**

Curation is essential for any data platform to maintain the quality of the data it provides. Today, more effective curation tools are often vital to keep up with the rapid growth of existing, maintenance-requiring databases and the amount of newly published information that needs to be surveyed. However, curation interfaces are often complex and challenging to be further developed. Therefore, opportunities for experimentation with curation workflows may be lost due to a lack of development resources or a reluctance to change sensitive production systems.

**Results:**

We propose a decoupled, modular and scriptable architecture to build new curation tools on top of existing platforms. Our architecture treats the existing platform as a black box. It, therefore, only relies on its public application programming interfaces and web application instead of requiring any changes to the existing infrastructure. As a case study, we have implemented this architecture in cmd-iaso, a curation tool for the identifiers.org registry. With cmd-iaso, we also show that the proposed design’s flexibility can be utilized to streamline and enhance the curator’s workflow with the platform’s existing web interface.

**Availabilityand implementation:**

The cmd-iaso curation tool is implemented in Python 3.7+ and supports Linux, macOS and Windows. Its source code and documentation are freely available from https://github.com/identifiers-org/cmd-iaso. It is also published as a Docker container at https://hub.docker.com/r/identifiersorg/cmd-iaso.

**Supplementary information:**

Supplementary data are available at *Bioinformatics* online.

## 1 Introduction

Improving the curation process on an existing data platform is often difficult. Curation workflows might be tightly coupled to the infrastructure, which increases the cost of any change. The platform might also no longer be technically maintained, its implementation might be outdated, or its original programmers might have left. In all of these cases, a curation tool that treats the underlying platform as a black box and only interacts with its existing application programming interfaces (APIs) would allow continuous expansions of the curation process without touching the underlying data platform.

We have used the identifiers.orgregistry data platform as a case study for this application note. Identifiers.org provides stable, globally unique identifiers for hundreds of data collections, mainly in the Life Sciences domain (Juty et al., 2013). To ensure these identifiers can be translated to a working URL (Wimalaratne et al., 2018), its registry stores manually curated, high-quality metadata for all collections, which must be kept accurate and up to date. Previously, identifiers.org used the HTTP response code of regular ping requests to determine whether dataset providers were still working (Juty *et al.*, 2013). In case of failure, a curator would then still have to investigate the type of error manually. For instance, the curator would have to come up with and test out multiple different stable identifiers at several points in time to distinguish between a planned outage or an outdated URL. This existing process was not further automated because of the cost to change the infrastructure.

We have developed the application cmd-iaso as a case study of our decoupled curation tool architecture, which does not require any changes to the existing architecture, allowing for quicker prototyping of curation workflows. We designed cmd-iaso to help with the current curation workflows in identifiers.org and any future ones. The tool is run in two stages. First, cmd-iaso runs expensive and long-running data gathering and analysis tasks in the background without supervision from a human curator. For instance, cmd-iaso can observe the data providers identifiers.org lists over multiple days. Second, cmd-iaso allows the curator to perform interactive analysis of the collected data, and guides them through an augmented user-interface of identifiers.org to highlight any problems that have been identified.

## 2 Implementation

**Fig. 1.**
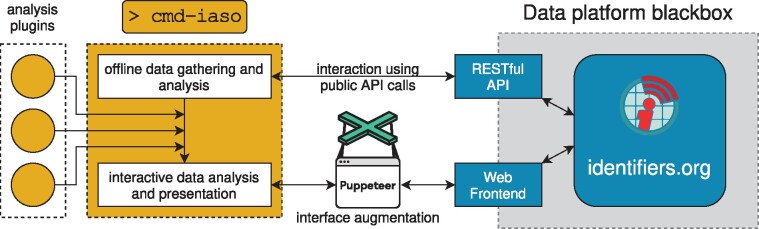
Overview of the decoupled software architecture of cmd-iaso

Figure 1 shows an overview of how we have implemented the proposed architecture. cmd-iaso is fully decoupled from identifiers.org, treats the platform as a black box and only communicates with it through its public APIs. In particular, technical knowledge of how the underlying data platform works is not required for implementing new curation workflows. As cmd-iaso is also designed as a modular tool, it can easily be extended with new analysis and curation workflows in an agile way. For instance, new data sources can easily be integrated into a workflow without changing any part of the underlying identifiers.org platform. To further reduce the technical barrier of prototyping extensions to the tool, cmd-iaso is written in the scripting language Python. Please refer to Supplementary Materials SIII and SIV for details on the implementation of cmd-iaso’s modular plugin system and an example analysis using the tool, respectively.

The most significant feature of cmd-iaso is its interactive curation workflow, during which the curator is guided through the identified issues. The tool can be run in a text-only terminal-based mode. However, cmd-iaso can best assist curation in its browser-based mode, in which it augments the existing web application of the platform. cmd-iaso uses pyppeteer, a Python port of the browser automation library Puppeteer. Puppeteer can launch or connect to a session of the Chrome browser and take complete control over it. For instance, the library can inject new information and control existing elements on any websites.

If cmd-iaso is run in its browser-based curation mode, it injects a control interface into identifiers.org’s website so that the curator can quickly jump between the problematic entries. It also automatically navigates to the corresponding page in the registry and augments it with an information overlay containing information about the issue, relevant hyperlinks and any proposed corrections. Please see Supplementary Materials SI and SII for a visualization of a typical curation session using cmd-iaso and more implementation details. It is worth emphasizing that the entire augmentation only occurs locally in the curator’s browser. This augmentation is the perfect example of how our proposed decoupled tool architecture can extend and improve the curator’s existing interaction with their platform.

## 3 Discussion

We have proposed a decoupled, modular and scriptable architecture for a curation tool, opening up the possibility for agile development and a diverse, easily maintainable set of plugins. As a case study, we have implemented this architecture in cmd-iaso, demonstrating the benefit of the proposed decoupled architecture. In particular, cmd-iaso’s flexible analysis plugin system is promising. The proposed modular architecture can even be viewed as a general and highly customizable curation toolbox, as it would simplify the integration and curation of different data platforms with various analysis methods.

So far, we have only tested the architecture in our case study of cmd-iaso. However, the proposed approach can be generalized to any data platform. Furthermore, the flexible architecture is very suitable to close collaboration between curators and developers. Specifically, we also envision a modern interpretation of the curators’ role, in which they have increasing ownership of and responsibility for the tools that support their curation workflows. The proposed architecture allows curators to be better equipped for the rapidly changing needs and magnitude of data in Life Sciences today (Tang et al., 2019).

## Funding

This work was supported by the European Molecular Biology Laboratory (EMBL) and the European Union’s Horizon 2020 research and innovation programme under [777523 FREYA].

*Conflict of Interest*: none declared.

## Supplementary Material

btab233_Supplementary_Data
